# Distalization of tibial tubercle osteotomy is not necessary for patients with recurrent patellar dislocation accompanied by patella alta and increased TT–TG distance

**DOI:** 10.1186/s12891-022-05779-8

**Published:** 2022-09-03

**Authors:** Kezhen Zhou, Pengchen Bai, Zhiwen Sun, Yanfeng Jia, Fei Wang, Xiaofeng Wang, Yingzhen Niu

**Affiliations:** Department of Orthopaedic Surgery, Third Hospital of Hebei Medical University, 139 Ziqiang Road, Shijiazhuang, 050051 Hebei People’s Republic of China

**Keywords:** Medial patellofemoral ligament, MPFL, Patellar instability, Tibial tuberosity transfer

## Abstract

**Background:**

The aim of this study is to determine whether distalization of the tibial tubercle is necessary for patients with recurrent patellar dislocation accompanied by patella alta and increased TT-TG.

**Methods:**

In this retrospective study, all 70 patients (70 knees) with recurrent patellar dislocation accompanied by TT–TG distance ≥20 mm and patella alta (CD-I ≥ 1.4) were surgically treated using MPFLR combined with medialization of the tibial tubercle or medialization and distalization of the tibial tubercle in the Third Hospital of Hebei Medical University between 2017 and 2019. 33 patients(33 knees) received MPFLR combined with medialization of the tibial tubercle (MPFLR + TTm group), 37 patients(37 knees) received MPFLR combined with medialization and distalization of the tibial tubercle (MPFLR + TTm-d group). Evaluation indicators included knee injury and osteoarthritis prognostic score (KOOS) and Kujala score evaluation, congruence angle (CA), patellar tilt angle (PTA), TT-TG distance, Blackburne-Peel index (BP-I), Caton-Deschamps index (CD-I).

**Results:**

A total of 70 knees (70 patients) with a mean follow-up time of 32 ± 6 months were evaluated in the present study. The postoperative, the PTA, CA, CD-I, BP-I, and TT-TG distance significantly improved in the two groups (*P* < 0.05), and there was no statistical difference between the two groups (>0.05). The KOOS and Kujala scores of the two groups at the last follow-up were significantly higher than the preoperative scores (*P* < 0.05), and there was no statistical difference between the two groups (*P*>0.05). No complications were noted in either group.

**Conclusion:**

For patients with recurrent patellar dislocation accompanied by increased TT-TG distance and patella alta, distalization is not needed and medialization is sufficient even in the presence of patella alta.

## Background

In a young, active patient population, recurrent patellar dislocation may be a major cause of persistent pain and functional limitation [1]. In the general population, 6 in 100,000 people have patellar dislocation [2–4]. Owing to the complexity of the pathogenic factors of the disease [5, 6], including patella alta, increased TT-TG distance, femoral trochlear dysplasia, frontal and torsional alignment, and patellofemoral ligament injury, the treatment of patellar dislocation is a challenging task for many orthopedic surgeons. Different degrees of dysfunction in one or more of these structures are often the cause of patellar dislocation. Recurrence rates have been reported to be as high as 15-44% after conservative treatment, often requiring surgery treatment [5, 6]. Surgical treatment methods can be roughly divided into soft tissue balance and bone balance surgery [7].

Currently, MPFL reconstruction (MPFL), either alone or in combination with procedures such as femoral distal derotation osteotomy (DDFO), tibial tubercle osteotomy (TTO), etc. has emerged as an effective treatment for recurrent patellar instability [8–10]. Isolated MPFLR sometimes does not achieve good results, especially in the case of the patella alta and increased distance, because it only addresses soft tissue abnormalities and not bony tissue abnormalities [11, 12]. The tibial tuberosity transfer has always been regarded as the standard procedure, which restores the relationship between the trochlear groove and the tibial tubercle as well as the height of the patella, achieving satisfactory clinical results [13]. For patients with recurrent patellar dislocation accompanied by patella alta and increased TT-TG distance, MPFLR combined with tibial tubercle transfer achieved a higher satisfaction rate [11]. Although, there are various types of tibial tubercle transfer operations [11]. For patients with recurrent patellar dislocation accompanied by patella alta and increased TT-TG distance, MPFLR combined with medialization and distalization of the tibial tubercle is one of the surgical methods in the past [14, 15]. Studies [14, 15] have shown that MPFLR can reduce or restore the height of the patella to normal levels, which has attracted attention. Therefore, the purpose of this study is to evaluate the necessity of distalization of the tibial tubercle in patients with recurrent patellar dislocation accompanied by increased TT-TG distance and patella alta.

We hypothesize that medialization of the tibial tubercle in patients with recurrent patellar dislocation accompanied by increased TT-TG and patella alta is sufficient.

## Materials and methods

### Subject

From 2017 and 2019, a total of 80 patients (86 knees) were enrolled in this retrospective study, who underwent the MPFLR combined with medialization of tibial tubercle or medialization and distalization of tibial tubercle at our hospital.

The inclusion criteria were: (1) recurrent patellar dislocation, (2) TT–TG distance ≥20 mm and patella alta [the CD-I ≥ 1.4], (3) failed nonoperative treatment, (4) were >18 years old at surgery, (5) followed up at a minimum of 2 years postoperatively; The exclusion criteria included: (1) other knee surgery, (2) ligament injuries other than the patellofemoral ligament, (3) high grade trochlear dysplasia (Dejour type C or greater), (4) history of previous knee surgery, (5) rheumatoid arthritis, (6) incomplete imaging examination.

Based on these criteria, 70 patients (70 knees) with recurrent patellar dislocation accompanied by TT–TG distance ≥20 mm and patella alta (the CD-I ≥ 1.4) were left in this study. The 33 patients (33 knees) in MPFLR + TTm group (MPFLR combined with medialization of the tibial tubercle) and the 37 patients (37 knees) in MPFLR + TTm-d group (MPFLR combined with medialization and distalization of the tibial tubercle). Patients’ radiological, and functional assessments were collected preoperatively and at follow-ups. Patients were followed up at a minimum of 2 years postoperatively with a mean follow-up of 32 ± 6 months. The clinical outcome was measured by means of two subjective questionnaires, KOOS and Kujala score evaluation. The demographic data of all patients are presented in Table 1. This study was approved by the Academic Ethics Committee of the Third Hospital of Hebei Medical University, and all patients gave their informed consent. All methods were carried out in accordance with relevant guidelines and regulations.

**Table 1:** Demographic data

	MPFLR + TTm group	MPFLR + TTm-d group	***P***-value
Gender, male/female	11/22	24/13	n.s.
BMI	24 ± 5	23 ± 4	n.s.
Age at surgery (years)	24 ± 6	23 ± 6	n.s.
Trochlear dysplasia			
NO	7	9	
A	14	18	
B	12	10	

*BMI* Body mass index

### Surgical technique

All operations were performed by a senior physician. First of all, arthroscopic exploration was carried out by conventional infrapatellar and extrapatellar approaches, and related intra-articular lesions (cartilage and meniscus damage, etc.) were explored. After the exploration, it was decided whether to perform lateral retinaculum release according to the results of the patellar push-in test.

#### MPFLR + TTm group

**Fig. 1:**
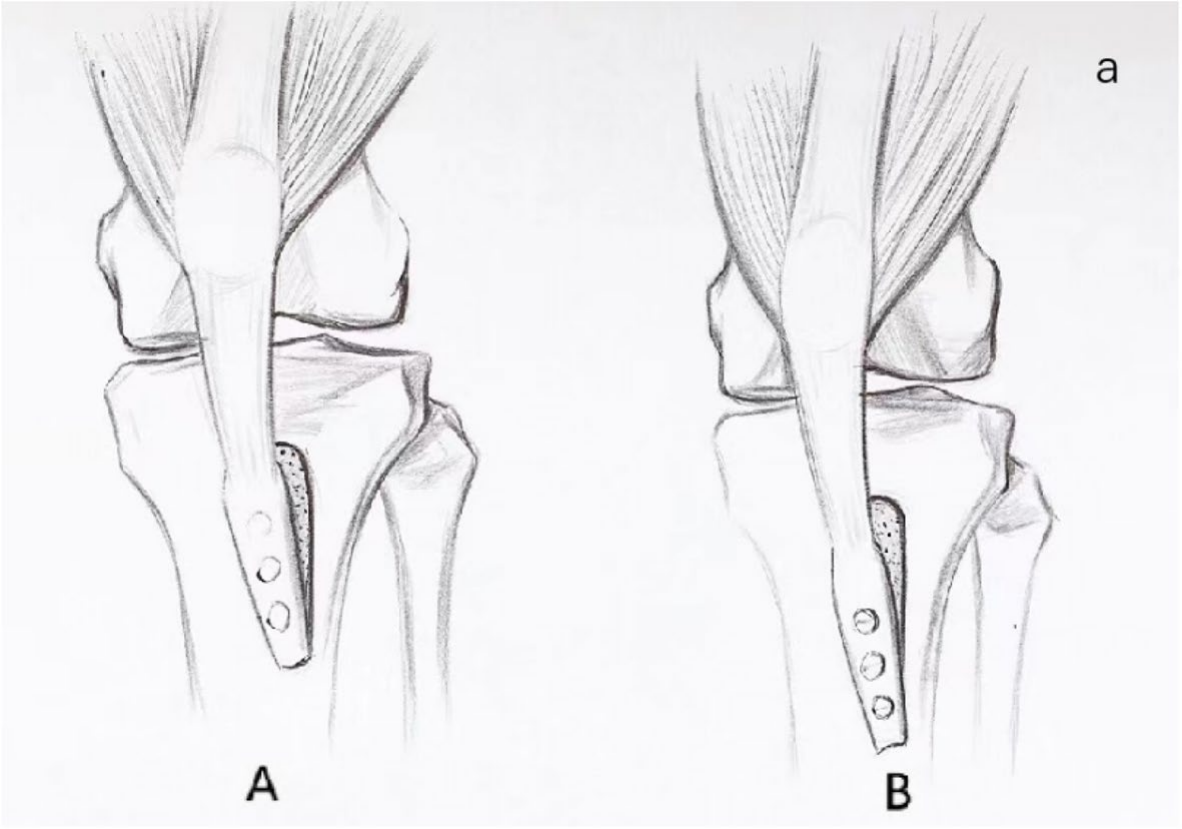
Schematic diagram of the surgical plan. **A** Medialization of the tibial tubercle. **B** Medialization and distalization of the tibial tubercle

The osteotomy plane starts from the patellar tendon at the tibial insertion point and descends 5 cm, and the L-shaped osteotomy line is determined. The distal end of the osteotomy block gradually thins and narrows. The soft tissue of the distal portion of the tibial tuberosity is preserved to be continuous with the tibia, and this soft tissue can act as a hinge. The tibial tubercle was moved medially according to the distance measured before the operation, to ensure that the TT-TG was 12 mm, and temporarily fixed with a bone round pin; the patellofemoral joint relationship and the patella movement track were observed under arthroscopy. The tibial tubercle was fixed with three diameter metal cortical bone screws, and the screws were perpendicular to the osteotomy plane to press the fracture surface. The head end of the screw should pass through the contralateral cortex to confirm the position of the tibial tubercle and the screw under intraoperative C-arm fluoroscopy (Fig. 1).

#### MPFLR + TTm-d group

The same as MPFLR + TTm group, the calculated value before the operation will move the bone block down and inward to ensure that the TT-TG is 12 mm, and at the same time, the high patella is corrected so that the CD-1 is 1 (Fig. 1).

#### MPFLR

Use the original tibial tubercle surgical incision to find the pes anserinus, and remove the gracilis tendon with a tendon extractor. All patients underwent anatomical double-bundled patellofemoral ligament reconstruction. The femoral insertion was located at the midpoint of the adductor tubercle and medial epicondyle of the femur. The medial patellofemoral ligament (MPFL) femoral insertion was determined by intraoperative fluoroscopy using the Schoettle point. The fixation method on the patellar side is to use 2 suture anchors. The femoral side is fixed with an absorbable screw.

After surgery, the joint cavity was flushed, a catheter was placed for drainage, the incisions were sutured in turn, and sterile dressings and elastic bandages were applied for pressure dressing.

### Data collection

The KOOS and Kujala questionnaire were analyzed preoperatively and at last follow-up. Radiological examination, including lateral radiograph of the knee joint with the knee flexed 30°, transverse plane of the knee joint with the knee flexed 30°, and Knee CT scans in the extended position. Radiological assessments include the CD-I, BP-I, CA, PTA, TT-TG distance, and the Dejour classification for trochlear dysplasia. To determine test-retest reliability, 35 patients were randomly selected from both groups (*n* = 70). Congruence angle was measured by the same examiner with a one-week interval between measurements.

#### PTA

**Fig. 2:**
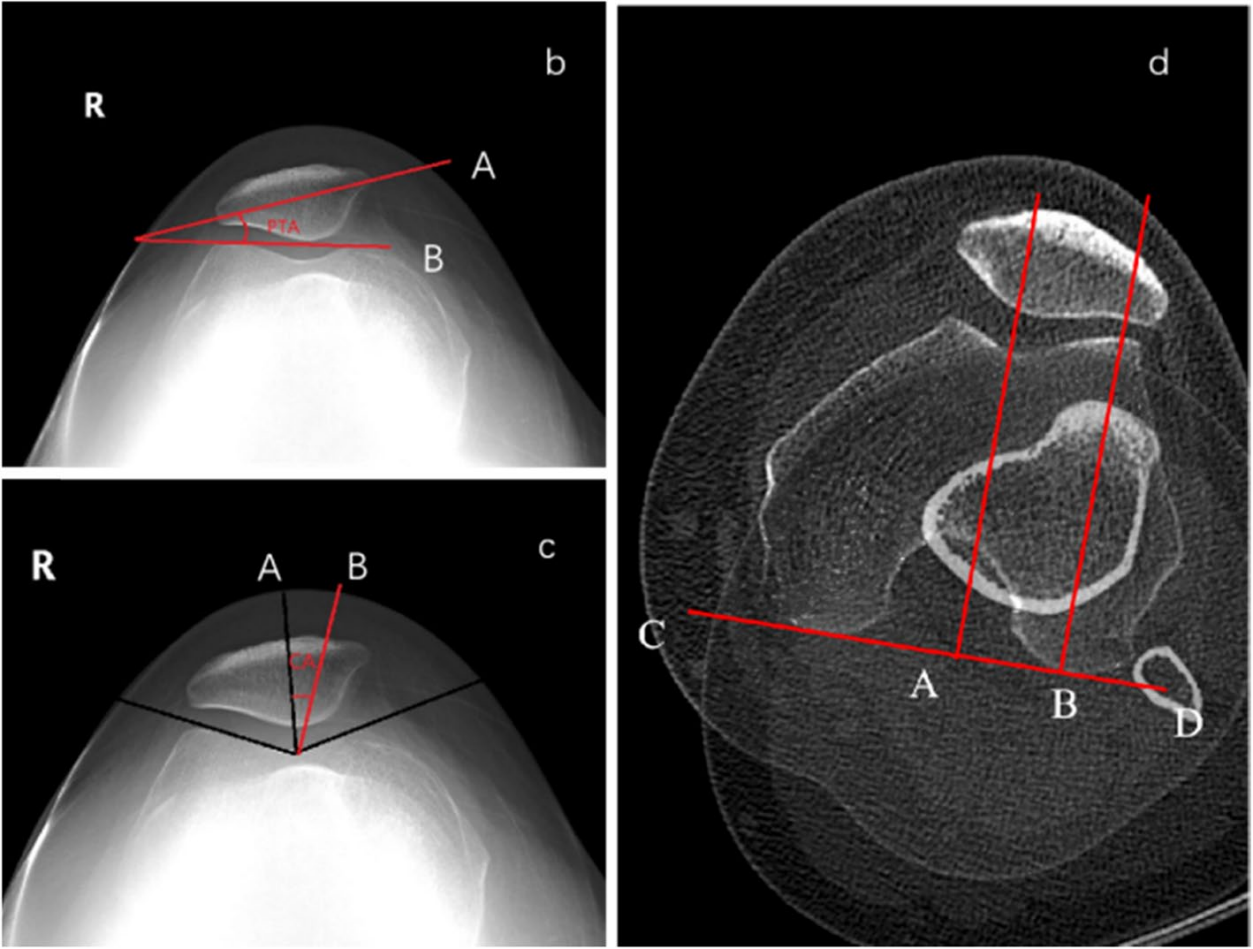
Schematic diagram of measurement of PTA, CA, and TTTG. **b** PTA: Patellar Tilt Angle; The angle between the line connecting the highest points of the medial and lateral condyles of the femur (**B**) and the line extending the maximum transverse diameter of the patella (**A**) is the PTA. **c** CA: Congruence Angle; Line A is the angle bisector of the trochlear groove of the femur, line B is the line connecting the deepest point of the trochlear groove of the femur and the lowest point of the patella, and the angle between the two lines of AB is CA. **d** TT-TG: Tibial Tuberosity-Trochlear Groove; Plane 1: the roof of the femoral intercondylar fossa is the “Roman arch”. Plane 2: near the proximal end of the tibial tubercle. Overlap the two planes. The line (CD) connecting the posterior femoral condyle is the marker point, marking the lowest point of the femoral trochlea and the midpoint of the tibial tubercle. Project them on the reference line respectively, and measure the distance between AB. It’s TT-TG

Measured on the patella axial radiograph with the knee flexed 30°. The angle is between the line connecting the posterior condyle of the femur and the line connecting the medial and lateral maximum transverse diameter of the patella (Fig. 2).

#### Ca

Measured on the patella axial radiograph with the knee flexed 30°. The angle is formed by the angle bisector of the trochlear groove of the femur and the line connecting the lowest point of the trochlear notch and the lowest point of the median ridge of the patella (Fig. 2).

#### TT-TG distance

Measured on CT of the knee joint in extension. Taking the posterior condyle of the femur as the reference line, the lowest point of the trochlear groove of the femur and the midpoint of the tibial tubercle are projected on the reference line respectively, and the distance between these two points is the TT-TG distance. Normal value: 8-10 mm (Fig. 2).

#### Cd-I

**Fig. 3:**
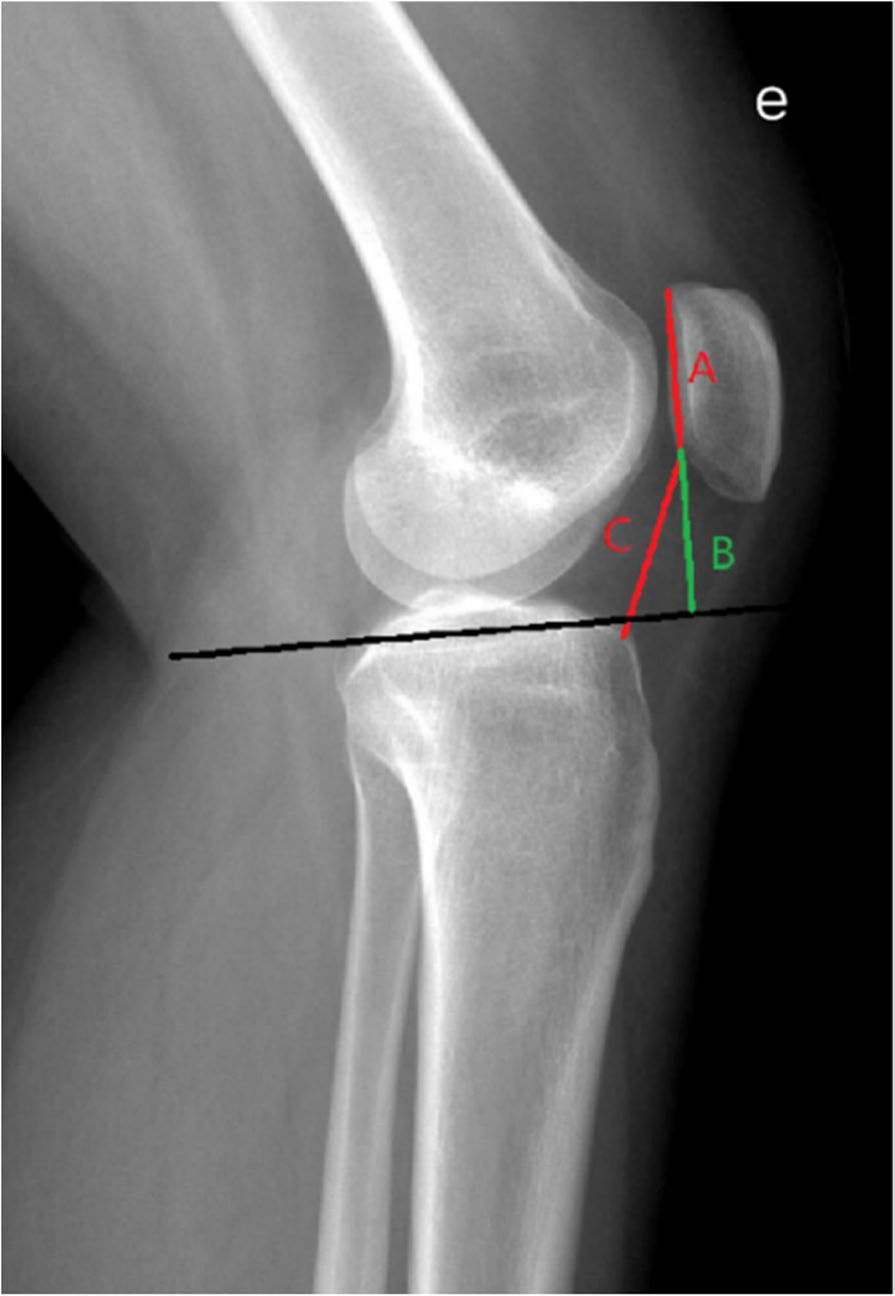
Radiographic patellar height indices. Caton-Deschamps index, The ratio of the distance from the lowest point of the patellar articular surface to the upper corner of the tibial plateau (**C**) to the length of the patellar articular surface (**A**). Blackburne-Peel index, The ratio of the vertical distance (**B**) from the lowest point of the patellar articular surface to the tibial plateau to the length of the patellar articular surface (**A**)

Measured on lateral radiographs with the knee flexed 30°.The ratio of the shortest distance from the lowest point of the patellar articular surface to the anterior superior angle of the tibial plateau contour to the length of the patellar articular surface. Patella alta>1.2 (Fig. 3).

#### BP-I

Measured on lateral radiographs with the knee flexed 30°. Measure the ratio of the vertical distance from the lower edge of the patellar articular surface to the tibial plateau to the length of the patellar articular surface. Patella alta>1.0 (Fig. 3).

### Statistical analyses

Before the investigation, the sample size was estimated with the KOOS as the primary variable. Means and standard deviations were used to describe continuous variables and proportions for categorical variables. Shapiro-Wilk test was used to determine date normality. A two-sample t-test was used to compare the differences between the two groups. Differences between pre-and post-operative were calculated using matched t-test. The Chi-square test was used to compare categorical variables between groups. All statistical analyses were performed using SPSS software (version 26, IBM Corp, Armonk, N). When the *p*-value <0.05, it was considered statistically significant. For a confidence level of 95% (α = 0.05) and power (1 − β) of 80%, a sample size of 29 patients per group was required.

## Results

A total of 70 knees (70 patients) with a mean follow-up time of 32 ± 6 months were evaluated in the present study.

### Clinical outcomes

Between pre-and postoperative period, a significant improvement in KOOS and Kujala functional scores were observed (*P* < 0.05). There was no significant difference between the two groups before and after surgery (*P* > 0.05) (Table 2).

**Table 2:** Comparison of clinical outcomes between the two study groups

	MPFLR + TTm group		MPFLR + TTm-d group		***P*** value
CD-I					
Pre	1.43 ± 0.03		1.44 ± 0.04		n.s.
Post	1.11 ± 0.09^a^		1.07 ± 0.08^a^		n.s.
BP-I					
Pre	1.27 ± 0.03		1.28 ± 0.04		n.s.
Post	0.99 ± 0.08^a^		0.94 ± 0.07^a^		n.s.
CA (°)					
Pre	18.6° ± 2.9°		18.7° ± 3.2°		n.s.
Post	4.9° ± 1.1°^a^		4.1° ± 1.5°^a^		n.s.
PTA (°)					
Pre	26.7° ± 2.9°		25.7° ± 3.2°		n.s.
Post	11.2° ± 1.4°^a^		11.6° ± 1.3°^a^		n.s.
TT-TG (mm)					
Pre	21.7 ± 1.4		22.1 ± 1.6		n.s.
Post	12.4 ± 1.1^a^		11.5 ± 1.3^a^		n.s.
KOOS					
Pre	61.4 ± 4.1		62.4 ± 5.4		n.s.
Post	82.3 ± 2.3^a^		81.8 ± 2.2^a^		n.s.
Kujala score					
Pre	61.2 ± 4.9		61.3 ± 5.6		n.s.
Post	80.4 ± 4.6^a^		80.2 ± 3.5^a^		n.s.

*KOOS* Knee injury and osteoarthritis outcome score, *CA* congruence angle, *PTA* patellar tilt angle, *TT–TG distance* tibial tuberosity–trochlear groove distance

^a^ There are statistical differences pre and post-surgery, *p*<0.05

### Radiographic outcomes

The mean pre-and postoperative patellar height measurements with the BP-I and CD-I in the two groups are listed in Table 2. All demonstrated a statistically significant reduction in patellar height.

The CD-I and BP-I of MPFLR + TTm group mostly decreased. 30.3% of patients (10 of 33) met the criteria for patella alta postoperative, which represents a 69.7% (23 of 33) correction to the reference range. The CD-I and BP-I of MPFLR + TTm-d group mostly decreased. 10.8% of patients (4 of 37) met the criteria for patella alta postoperative, which represents an 89.2% (33 of 37) correction to the reference range (Table 3).

**Table 3:** Changes in patella height in patients who did not return to normal patella height after surgery

		Pre	Post	*P* value
MPFLR + TTm group (*n* = 10)	CD-I	1.43 ± 0.03	1.25 ± 0.05	<0.05
	BP-I	1.27 ± 0.03	1.11 ± 0.05	<0.05
MPFLR + TTm-d group (*n* = 4)	CD-I	1.41 ± 0.01	1.24 ± 0.01	<0.05
	BP-I	1.25 ± 0.01	1.10 ± 0.01	<0.05

In terms of PTA, CA, and TT-TG, the mean value of all patients improved significantly after surgery (*P*<0.05), and there was no statistical difference between the two groups (*P*>0.05) (Table 2).

### Complications

During the follow-up period, there were no complications such as subluxation, dislocation, incision infection, knee stiffness, osteotomy nonunion, superior tibia fracture, and vascular nerve injury in the two groups.

## Discussion

The main finding of the current study was no significant difference in postoperative outcomes between the two combined therapeutic protocols. And in the treatment of patellar dislocation with increased TT–TG distance and patella alta, both surgical protocols achieved good clinical outcomes.

Increased TT-TG and Patella alta have long been considered a risk factor for patellofemoral instability, and many patients meet the criteria for patella alta and increased TT–TG [16, 17]. TT-TG distance affects the involution relationship of the patellofemoral joint [16, 17]. When the TT-TG distance is too wide, the lateral sagittal force on the patella increases, resulting in patellar dislocation [15]. When the patella is in a high position, the time when the patella enters the trochlear groove is delayed or the patella even is unable to enter the trochlear groove, which reduces the effect of the trochlear groove to limits the lateral translation of the patella and increases the risk of patellar dislocation [18–20]. Additionally, patella alta has been noted to be a risk factor for recurrent instability after they undergo conservative treatment or isolated MPFLR. According to Mulliez et al., there were significant differences in clinical outcomes of MPFLR with or without tibial tubercle transfer, and the procedure of tibial tubercle transfer resulted in better clinical outcomes in selected populations. However, some studies [15] have also pointed out that MPFLR combined with tibial tubercle transfer has a deteriorating effect on cartilage. The indications for tibial tubercle transfer should be carefully evaluated. The effect of distalization on cartilage deterioration in the mid-to long-term remains unclear in the population of this study.

The choice of treatment options for patella alta is difficult. Some surgeons [21] believe that even in patients with patella alta or increased TT-TG distance without any additional bony surgery, isolated MPFLR can achieve better clinical outcomes without redislocation. However, these are retrospective studies based on small samples. There are also studies showing that for patients with patella alta and increased TT-TG, MPFLR combined with the medialization and distalization of the tibial tubercle has been used, and good results have been achieved [22, 23]. So far, there is no clear treatment plan for patella alta. In our study, both surgical protocols were shown to improve functional scores while improving patella alta. Second, we also demonstrated that even if orthopedic surgery appears to be more invasive, it does not affect the patient’s good clinical outcomes.

In this study, In MPFLR + TTm group, the patellar height did not return to the normal range in 10 patients after surgery, and the CD-I at the last follow-up was 1.25 ± 0.05. In MPFLR + TTm-d group, the patellar height did not return to the normal range in 4 patients after surgery, and the CD-I at the last follow-up was 1.24 ± 0.01. Although the postoperative patellar height of these patients did not return to the normal range, postoperative functional scores were improved compared with preoperative. Besides, no complications occurred during the follow-up in both group. Therefore, we deem that mild patella alta does not affect quality-of-life outcomes. This finding is consistent with previous research [24, 25]. Therefore, the Surgical protocol of MPFLR + TTm group is sufficient for the patients with patella alta and increased TT–TG distance. These findings are important for MPFLR surgeons because improved patella alta in the absence of tibial tubercle distalization may allow for the avoidance of an unnecessary additional surgical procedure.

Several reasons may account for this phenomenon. In a previous study [26], patella alta was improved after patellar ligament reconstruction alone. But in their study, the location of the femoral insertion of the patellofemoral ligament is at the distal end of the epiphysis. With the increase of age, the growth of the epiphysis begins, and finally, the position of the patella decreases. However, in our study, adults’ epiphysis has all been closed. Besides, it is not clear whether the topic of patella alta is a risk factor or a result of patellofemoral dislocation. Fabricant et al. [26] speculate that this may be due to the MPFL rupturing itself. It is known that the MPFL is an origin on the patella more proximal than the distal insertion on the femur. Therefore, when the patellofemoral ligament is injured, the position of the patella may be shifted upwards, then the patella alta is measured on the injury radiographs. However, many studies have shown good clinical outcomes after patellar ligament reconstruction alone, without the reduction in the patellar height [27].

Several limitations should be considered for this study. One is that we ruled out severe trochlear dysplasia. Studies have shown that trochlear dysplasia of the femur has a negative impact on the prognosis of MPFLR with or without tibial tubercle transfer [28]. Second, the follow-up period is short, and long-term follow-up and large-scale clinical trials should be conducted in the future to validate our findings. Third, our surgery is MPFLR with tibial tubercle transfer, so it is unclear whether the decreased patella position is caused by the MPFLR or caused by the tibial tubercle transfer. Lastly, our article does not consider the influence of frontal and torsional Alignment, which are bony factors that extremely influence the tracking of the patella.

## Conclusion

For patients with recurrent patellar dislocation accompanied by increased TT-TG distance and patella alta, distalization is not needed and medialization is sufficient even in the presence of patella alta.

## Data Availability

The datasets used during the current study are available from the corresponding author on reasonable request.

## Abbreviations

TT-TG: Tibial Tuberosity-Trochlear Groove
MPFLR: Medial Patellofemoral Ligament Reconstruction
TTm: Medialization of the tibial tubercle
TTm-d: Medialization and distalization of the tibial tubercle
TTO: Tibial tubercle osteotomy
KOOS: Knee injury and Osteoarthritis prognostic Score
CA: Congruence Angle
PTA: Patellar Tilt Angle
CD-I: Caton-Deschamps index
BP-I: Blackburne-Peel index
